# Retinal Artificial Intelligence‐based Model Identifies Non‐demented Elderly Subjects at Risk of Alzheimer's Disease

**DOI:** 10.1002/alz70856_098859

**Published:** 2025-12-24

**Authors:** Anran Ran, Yuen Tung Ng, Bonnie Y.K. Lam, Huijing Zheng, Lisa W.C. Au, Alexander Y.L. Lau, Alan H.F. Lam, Xiaoyan Hu, Ha Ying Chiu, Ho Ko, Carol Y Cheung, Vincent C.T. Mok

**Affiliations:** ^1^ Department of Ophthalmology and Visual Sciences, The Chinese University of Hong Kong, Hong Kong SAR, NA, Hong Kong; ^2^ Lam Kin Chung. Jet King‐Shing Ho Glaucoma Treatment and Research Centre, The Chinese University of Hong Kong, Hong Kong SAR, Hong Kong; ^3^ Division of Neurology, Department of Medicine and Therapeutics, The Chinese University of Hong Kong, Hong Kong SAR, Hong Kong; ^4^ Gerald Choa Neuroscience Institute, The Chinese University of Hong Kong, Hong Kong SAR, Hong Kong; ^5^ Li Ka Shing Institute of Health Sciences, Faculty of Medicine, The Chinese University of Hong Kong, Hong Kong SAR, Hong Kong; ^6^ Margaret K. L. Cheung Research Centre for Management of Parkinsonism, Faculty of Medicine, The Chinese University of Hong Kong, Hong Kong SAR, Hong Kong; ^7^ Lau Tat‐Chuen Research Centre of Brain Degenerative Diseases in Chinese, Faculty of Medicine, The Chinese University of Hong Kong, Hong Kong SAR, Hong Kong; ^8^ Department of Mechanical and Automation Engineering, The Chinese University of Hong Kong, Hong Kong SAR, Hong Kong; ^9^ Charles K Kao Foundation for Alzheimer's disease, Hong Kong SAR, Hong Kong; ^10^ Department of Ophthalmology and Visual Sciences, The Chinese University of Hong Kong, Hong Kong SAR, Hong Kong

## Abstract

**Background:**

RetinAD is a validated deep learning model for differentiating between Alzheimer's disease (AD) dementia and cognitively unimpaired subjects based on analyzing retinal photographs. Since certain AD‐related retinal changes (e.g., microvasculopathy) may start to develop years to decades before the onset of cognitive symptoms, we hypothesized that RetinAD may also identify retinal microvasculopathy among non‐demented elderly subjects. We aimed to compare measures of retinal vessel network between “positive” and “negative” cases as classified by RetinAD among elderly non‐demented elderly subjects.

**Method:**

We recruited community subjects who were participants in the **BEAT AD** (**B**rain Health **E**ducation **A**nd **T**ailor‐made Measures for Prevention of **A**lzheimer's **D**isease) service programme in Hong Kong. This programme invites non‐demented community dwelling subjects (59‐80 years old) with subjective cognitive decline (SCD). It assesses their cognitive performances using Montreal Cognitive Assessment‐5 minutes (MoCA‐5) and on their control in the modifiable risk factors of AD. It also provides tailor‐made recommendation for the subjects of how to optimize those risk factors that are not well controlled. We obtained fundus pictures using the Topcon NW500 non‐mydriatic retinal camera. We classified subjects into “positive” or “negative” using RetinAD. We conducted quantitative measurements of retinal vessels using the Singapore I Vessel Assessment (SIVA) software.

**Result:**

Among the 187 recruited subjects with SCD, 29 (15.5%) and 158 (84.5%) subjects were classified as “positive” and “negative”, respectively. Subjects who were classified as “positive” were older (mean age 71.21 versus [vs] 67.59; *p* = <0.01) than those who were classified as “negative”. There was no significant difference in MoCA scores between “positive” (22.79) and “negative” subjects (23.74, *p* = 0.28). Analysis of the retinal vessel network showed that “positive” subjects had a significantly higher branching coefficient arterioles (1.64 vs 1.49) and branching coefficient venules (1.49 vs 1.32) than that of “negative” subjects. The difference remained significant (*p* = 0.033) for the branching coefficient venules after being adjusted to age and mean arterial pressure.

**Conclusion:**

RetinAD identified non‐demented elderly who had worse retinal microvasculopathy and biologically older brains. Findings suggested that RetinAD may be able to identify elderly subjects who are at risk of developing AD dementia in the future.

